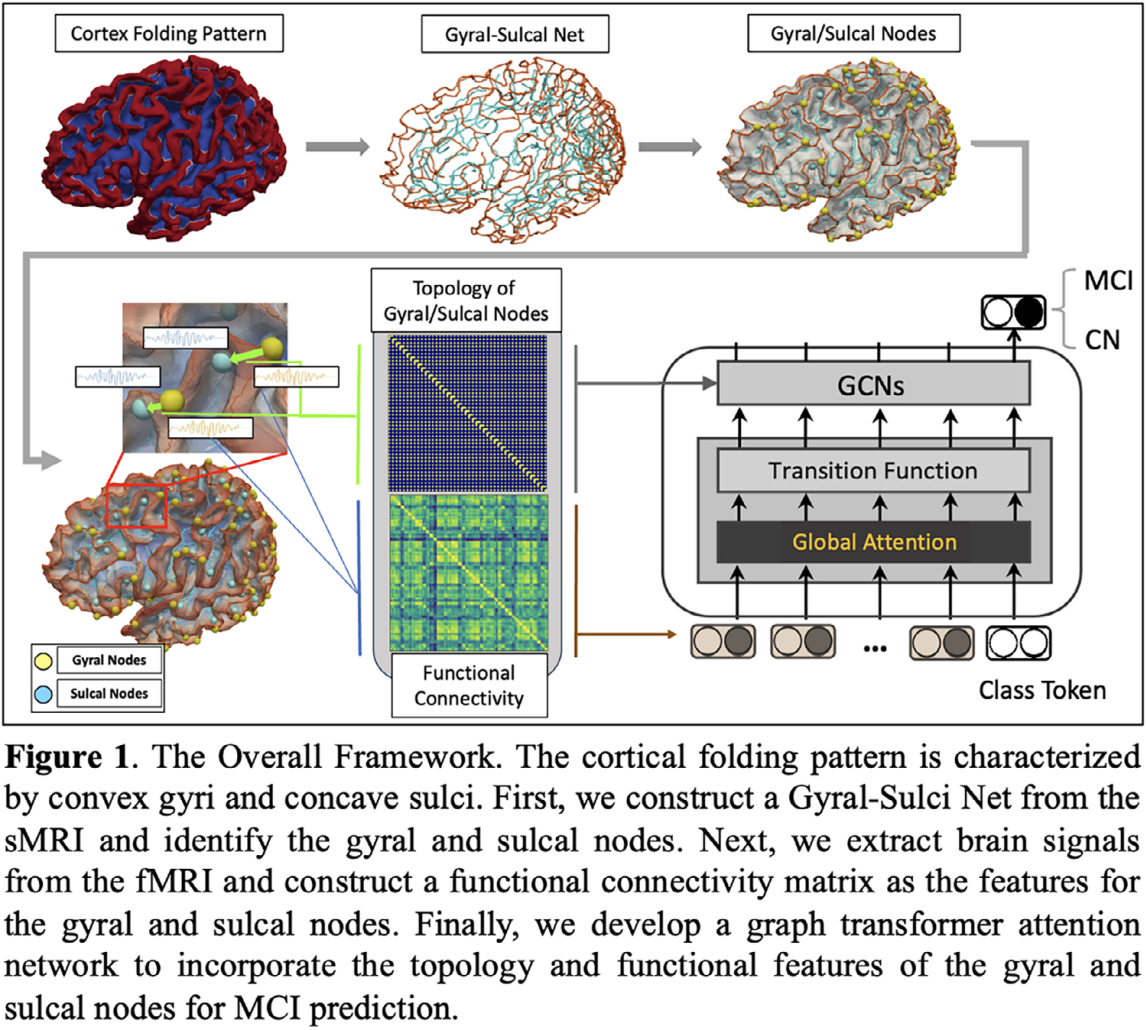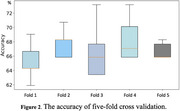# Gyral‐Sulcal Net: A Novel Brain Network Representation for Mild Cognitive Impairment Classification

**DOI:** 10.1002/alz70856_102417

**Published:** 2025-12-25

**Authors:** Yanjun Lyu, Xiaowei Yu, Michael Qu, Tong Chen, Nan Zhao, Xiang Li, Lu Zhang

**Affiliations:** ^1^ The University of Texas at Arlington, Arlington, TX, USA; ^2^ Mission San Jose High School, Fremont, CA, USA; ^3^ Indiana University Indianapolis, Indianapolis, IN, USA; ^4^ Massachusetts General Hospital and Harvard Medical School, Boston, MA, USA

## Abstract

**Background:**

Alzheimer's disease (AD), once established, cannot be reversed or cured. The diagnosis of mild cognitive impairment (MCI), often considered a precursor to AD, has become a more feasible goal. AD significantly affects individuals' brain structure and function. Therefore, it is crucial to incorporate features of brain structure and function simultaneously to provide a comprehensive diagnosis and effectively distinguish MCI from cognitively normal (CN) individuals. In this work, we propose a novel brain network representation, the Gyral‐Sulcal Net that provides finer‐scale brain structural landmarks along with corresponding functional features, to enhance the diagnostic accuracy of MCI from CN.

**Method:**

Our study utilized structural T1‐weighted structural MRI (sMRI) and functional MRI (fMRI) data from 126 MCI subjects and 141 CN subjects from the ADNI dataset. After the cortical surface reconstruction, we constructed the Gyral‐Sulcal Net from sMRI and identify gyral and sulcal nodes. The fMRI signals from gyral and sulcal nodes located at the corresponding region of interest (ROI) were extracted. We then calculated a functional connectivity matrix with such ROIs at dimensions of 88 × 88. For group prediction, we developed a graph transformer attention network, where the topology of gyral and sulcal nodes, and the functional connectivity matrix served as the feature input. The overall framework is illustrated in Figure 1.

**Result:**

The proposed method achieved promising results. We conducted 10 experiments and 5‐fold cross validation (best for each fold) for MCI/CN classification. We achieved a mean accuracy of 73.80% with a standard deviation of 2.97%. The cross‐validation results are shown in Figure 2.

**Conclusion:**

We proposed a novel brain Gyral‐Sulcal Net for MCI/CN classification. The Gyral‐Sulcal Net integrates the brain's two primary folding patterns, gyri and sulci, into a unified finer‐scale anatomical network. It effectively combines structural and functional information as the node features. Based on the Gyral‐Sulcal Net, we developed a novel graph transformer network to leverage Gyral‐Sulcal Net for MCI/CN classification. Our approach achieved promising results, highlighting the great potential of the Gyral‐Sulcal Net in improving diagnostic accuracy.